# Utility of routine post-admission testing for SARS-CoV-2 in a rehabilitation facility

**DOI:** 10.1017/ash.2024.14

**Published:** 2024-02-12

**Authors:** Victoria R. Williams, Larry Robinson, Morty Eisenberg, Kuldeep Virdi, Robert Kozak, Jerome A. Leis

**Affiliations:** 1 Infection Prevention and Control, Sunnybrook Health Sciences Centre, Toronto, ON, Canada; 2 Rehabilitation Sciences Institute, University of Toronto, Toronto, ON, Canada; 3 Shared Hospital Laboratory, Sunnybrook Health Sciences Centre, Toronto, ON, Canada; 4 Division of Infectious Diseases, Department of Medicine, University of Toronto, ON, Canada

## Abstract

Asymptomatic screening for SARS-CoV-2 is recommended in healthcare settings during periods of increased incidence, yet studies in rehabilitation settings are lacking. Routine weekly post-admission asymptomatic testing in a rehabilitation facility offered marginal gain beyond syndromic and targeted unit testing and was not associated with a reduced risk of healthcare-associated COVID-19.

Asymptomatic and pre-symptomatic SARS-CoV-2 infected patients are a potential source of transmission in healthcare settings.^1^ The Centers for Disease Control recommends SARS-CoV-2 testing for symptomatic patients, and asymptomatic testing at the time of admission, following exposure and in the event of suspected health-associated transmission but do not address routine asymptomatic post-admission screening.^2^ In one study in an acute care hospital, introduction of a 7-day recurring asymptomatic testing protocol led to an increase in detection of COVID-19 cases.^3^

Rehabilitation facilities are conducive to respiratory virus transmission due to the patient population and their mobilization in shared spaces.^4^ We hypothesized that routine weekly asymptomatic testing for SARS-CoV-2 in a rehabilitation hospital experiencing frequent COVID-19 outbreaks, may reduce the burden of healthcare-associated COVID-19.

We performed an uncontrolled before-after intervention study comparing baseline (April 1, 2021 to March 31, 2022) and intervention (April 1, 2022 to March 31, 2023) periods at a 154-bed rehabilitation hospital associated with a tertiary care acute care hospital. The facility has only 14(9%) single rooms and 50% of beds are in four-bed ward rooms. At baseline, mid-turbinate swabs were collected by nursing staff on all patients and tested for SARS-CoV-2 on admission (≤5 days), in response to the development of new symptoms consistent with COVID-19 infection (syndromic testing), following exposure to a patient with COVID-19 and as part of unit wide prevalence testing during suspected or confirmed outbreaks. The testing platform was a reverse-transcriptase polymerase chain reaction (RT-PCR) assay performed in a laboratory affiliated with our healthcare facilty.^5^

In addition to all pre-existing SARS-CoV-2 testing, the intervention introduced April 1, 2022, added routine weekly unit-wide asymptomatic prevalence screening whereby all patients on a given unit were tested on the same day. Prospective assessment was performed to identify cases of acute SARS-CoV-2 infection, defined as a patient testing positive for SARS-CoV-2 who had not been previously infected with COVID-19.^6^ Specifically, cases with documented COVID-19 within 90 days or with a cycle threshold (CT) value ≥28.0 who remained asymptomatic and had a repeat confirmatory swab that was negative or with CT value persistently ≥28.0 were considered recovered cases and excluded.^6^

The primary outcome was the yield of routine post-admission screening in comparison to the admission, outbreak-related and syndromic testing that was already in place. The secondary outcome was the incidence of healthcare associated-COVID-19 (HA-COVID) per 1,000 non-COVID-19 patient-days, defined as a COVID-19 case with a positive RT-PCR test or symptom onset ≥5 days after admission or <5 days following transfer to our organization’s associated acute care facility. Relative risk (RR) with 95% confidence intervals (CI) was used to compare percent positivity and HA-COVID incidence between the two periods. Infection prevention policies during both periods included universal masking for all healthcare workers (HCWs) and visitors, and transmission-based precautions (N95 respirator, eye protection, gowns and gloves) for all patients with suspected or confirmed COVID-19. Data was collected prospectively as part of routine surveillance and research ethics review was not required because the study met institutional criteria for exemption as quality improvement.

In total, 13529 tests for SARS-CoV-2 were performed throughout the study period, of which 13095 (96.8%) were eligible for inclusion while 434 tests were excluded including 350 (81%) from patients with recovered COVID-19 and 84 (19%) duplicates. The intervention period was associated with nearly double the number of eligible SARS-CoV-2 tests as compared to the baseline period (8513 vs. 4582). The number and yield of SARS-CoV-2 tests and the incidence of HA-COVID by rationale for testing is described in Table 1 and displayed by month in Figure 1. Overall, the percent positivity of all testing for SARS-CoV-2 increased during the intervention period (2.6% vs. 1.4%; RR 1.91, 95% CI 1.44–2.53; *p* < 0.001), which was driven by an increase in the percent positivity for admission screening (1.8% vs. 0.5%; RR 3.52, 95% CI 1.93–6.43; *P* < 0.001). In comparison, the percent positivity of post-admission asymptomatic testing during a suspect or confirmed outbreak period did not increase during the intervention period (4.9% vs. 3.2%; RR 1.5, 95% CI 0.68–3.41) nor did the percent positivity of testing patients with symptoms compatible with COVID-19 infection or following a known exposure (25.3% vs, 20.8%; RR 1.22, 95% CI 0.87–1.71). The addition of routine weekly testing had an extremely low yield of 0.2% (8/4022) while accounting for 47.2% (4022/8513) of all SARS-CoV-2 tests in the intervention period. The incidence of HA-COVID increased during the intervention period (4.1 vs. 1.3 per 1,000 non-COVID-19 patient-days; RR 3.15, 95% CI 2.37–4.23; *p* < 0.001). Detection of the additional HA-COVID cases occurred through syndromic/exposure testing (2.7 vs. 0.71 per 1,000 non-COVID-19 patient-days; RR 3.79, 95% CI 2.58–5.56; *p* < 0.001) and suspect/confirmed outbreak testing (0.65 vs. 0.15 per 1,000 non-COVID-19 patient-days; RR 4.35, 95% CI 1.91–9.91; *p* < 0.001).

**Table 1.** Characteristics of asymptomatic testing for SARS-CoV-2 in a rehabilitation setting and the incidence of healthcare-associated-COVID-19 before and after implementation of weekly prevalence screening

	Baseline (April 1, 2021–March 31, 2022)	Intervention (April 1, 2022–March 31, 2023)
Total No. of patients	1,678	1,811
Median age (IQR)	74(61–83)	74(62–83)
Male gender (%)	797(47.5)	872(48.2)
Patient days	47,444	48,437
Non-COVID-19 patient days	46,805	46,097
Median daily COVID-19 case patient census (IQR)	0(0–2)	3(1–8)
Total No. eligible SARS-CoV-2 tests	4,582	8,513
Median SARS-CoV-2 tests per patient admission (IQR)	2(2–3)	4(3–6)
No. (%) COVID-19 cases detected	62(1.4)	220(2.6)
No. of HA-COVID cases	61	189
Incidence per 1,000 non-COVID-19 patient days	1.3	4.1
Median age (IQR)	79(65–85)	76(63–85)
Male gender (%)	29(47.5)	99(52.4)
Median days from admission to detection (IQR)	13(9–24)	15(10–27)
Total No. admission SARS-CoV-2 tests	2575	3096
No. (%) COVID-19 cases detected	13(0.5)	55(1.8)
Total No. syndromic/exposure tests	159	487
No. (%) COVID-19 cases detected	33(20.8)	123(25.3)
No. of HA-COVID cases	33	123
Incidence per 1,000 non-COVID-19 patient days	0.71	2.7
Total No. suspect/confirmed outbreak tests	218	614
No. (%) COVID-19 cases detected	7(3.2)	30(4.9)
No. of HA-COVID cases	7	30
Incidence per 1,000 non-COVID-19 patient days	0.15	0.65
Total No. weekly prevalence tests	–	4022
No. (%) COVID-19 cases detected	–	8(0.2)
No. of HA-COVID cases	–	8
Incidence per 1,000 non-COVID-19 patient days	–	0.17
Total No. of COVID-19 outbreaks	6	12
No, outbreak associated COVID-19 cases	48	151

Note. IQR, interquartile rate; HA-COVID, healthcare-associated-COVID-19.

**Figure 1.**
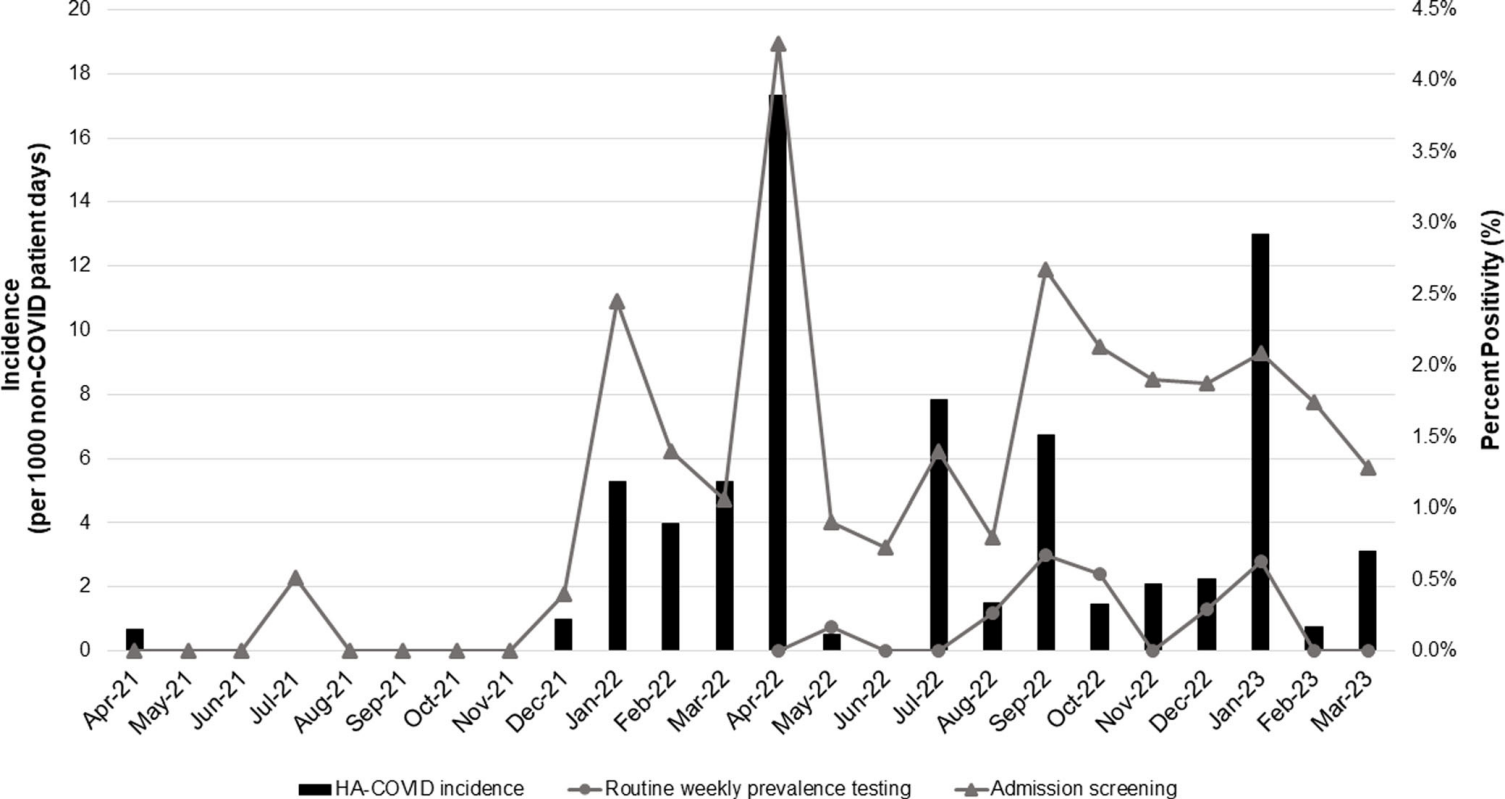
Monthly percent positivity of asymptomatic testing for SARS-CoV-2 between April 2021 and March 2023 as compared to the incidence of healthcare-associated-COVID-19 (HA-COVID) infection in a rehabilitation facility.

The marginal impact of routine SARS-CoV-2 post-admission testing in a rehabilitation facility experiencing significant healthcare-associated COVID-19 has several potential explanations. First, vigilant syndromic surveillance and contact tracing of confirmed cases of COVID-19 as well as prompt asymptomatic prevalence testing in response to nosocomial activity identified the majority of the cases that developed post-admission. A similar weekly testing protocol in an acute care hospital detected 40 cases of COVID-19 among admitted patients who tested negative for SARS-CoV-2 on admission, yet further investigation determined that 70% had documented symptoms of COVID-19 infection that should have been picked up on syndromic surveillance.^3^ The second possibility is that our testing protocol may have been too infrequent to contribute significantly to case detection. A simulation study of Chin et al reported that although once-weekly testing may be sufficient when community incidence is lower and additional interventions are in place, twice-weekly asymptomatic testing was required to reduce the number of cases of COVID-19 and prevent outbreaks in high-risk settings during periods of high community transmission.^7^ A separate modeling study in a mental health setting found that once weekly testing reduced risk of an outbreak by 49% while twice weekly reduced it by 67%.^8^

There are significant limitations to our study. First, it was a single center uncontrolled study in a rehabilitation facility and may not be applicable to other healthcare settings. However, given that post-admission asymptomatic screening was low yield in our facility with a majority of multi-bed rooms it may be even less useful in a rehabilitation hospital with newer infrastructure. Second, the increase in HA-COVID during the intervention period was likely related to the increased transmissibility associated with the Omicron variant throughout the intervention period as compared to ancestral SARS-CoV-2 variants circulating during the baseline period.^9,10^ These increased cases were detected through usual syndromic and investigation-driven surveillance and not through routine post-admission testing. Third, due to limited availability of testing during the study period, community positivity rates are not available for comparison. The increased admission testing positivity remains a surrogate for the higher community burden during the intervention period. Finally, we do not know if more frequent asymptomatic post-admission testing would reduce the risk of transmission in the rehabilitation setting. The resources required to sustain a program that routinely tests more than weekly made it unfeasible to implement.

In a rehabilitation setting, the addition of routine weekly asymptomatic prevalence testing of patients was of low yield when added to existing surveillance in place. These findings do not support routine weekly post-admission testing in rehabilitation.

## References

[1] He X , Lau EHY , Wu P , et al. Temporal dynamics in viral shedding and transmissibility of COVID-19. Nat Med 2020;26:672–675. doi: 10.1038/s41591-020-0869-5. 32296168

[2] CDC. Interim infection prevention and control recommendations for healthcare personnel during the Coronavirus disease 2019 (COVID-19) pandemic, 2022. https://www.cdc.gov/coronavirus/2019-ncov/hcp/infection-control-recommendations.html

[3] Smith L , Pau S , Fallon S , et al. Impact of weekly asymptomatic testing for severe acute respiratory coronavirus 2 (SARS-CoV-2) in inpatients at an academic hospital. Infect Control Hosp Epidemiol 2023;44:99–101. doi: 10.1017/ice.2021.384. 34446117 PMC8446587

[4] Corpus C , Williams V , Salt N , et al. Prevention of respiratory outbreaks in the rehabilitation setting. BMJ Open Qual 2019;8:e000663. doi: 10.1136/bmjoq-2019-000663. PMC6797241 31673641

[5] Kandel CE , Young M , Serbanescu MA , et al. Detection of severe acute respiratory coronavirus 2 (SARS-CoV-2) in outpatients: A multicenter comparison of self-collected saline gargle, oral swab, and combined oral-anterior nasal swab to a provider collected nasopharyngeal swab. Infect Control Hosp Epidemiol 2021;42:1340–1344. doi: 10.1017/ice.2021.2. 33436122 PMC7870921

[6] Williams VR , Chan CK , Chan AK , Kozak R , Leis JA. Impact of universal admission testing for severe acute respiratory coronavirus 2 (SARS-CoV-2) in era of the Omicron variant. Infect Control Hosp Epidemiol 2023;44:102–105. doi: 10.1017/ice.2022.273. 36651289

[7] Chin ET , Huynh BQ , Chapman LAC , Murrill M , Basun S , Lo NC. Frequency of routine testing for COVID-19 in high-risk healthcare environments to reduce outbreaks. Clin Infect Dis 2021;73:e3127–e3129. doi: 10.1093/cid/ciaa1383. 33570097 PMC7797732

[8] Litwin T , Timmer J , Berger M , Wahl-Kordon A , Muller MJ , Kreutz C. Preventing COVID-19 outbreaks through surveillance testing in healthcare facilities: a modeling study. BMJ Infect Dis 2022;22:105. doi: 10.1186/s12879-022-07075-1. PMC8800405 35093012

[9] Williams VR , Tan C , Kozak R , Leis JA. Incidence and outcomes of hospital-associated coronavirus disease 2019 (COVID-19) before and after emergence of the severe acute respiratory coronavirus 2 (SARS-CoV-2) omicron variant. Infect Control Hosp Epidemiol 2023. online ahead of print. doi: 10.1017/ice.2023.29. PMC10587372 36876324

[10] Klompas M , Padolfi MC , Nisar AB , Baker MA , Rhee C. Association of omicron vs wild-type SARS-CoV-2 variants with hospital-onset SARS-CoV-2 infections in a US regional hospital system. JAMA 2022;328:296–298. doi: 10.1001/jama.2022.9609. 35704347 PMC9201738

